# Comprehensive metabolic profiling of chronic low-grade inflammation among generally healthy individuals

**DOI:** 10.1186/s12916-017-0974-6

**Published:** 2017-11-30

**Authors:** Maik Pietzner, Anne Kaul, Ann-Kristin Henning, Gabi Kastenmüller, Anna Artati, Markus M. Lerch, Jerzy Adamski, Matthias Nauck, Nele Friedrich

**Affiliations:** 1 Institute of Clinical Chemistry and Laboratory Medicine, University Medicine Greifswald, Ferdinand-Sauerbruch-Str. NK, 17475 Greifswald, Germany; 2 DZHK (German Centre for Cardiovascular Research), partner site Greifswald, Greifswald, Germany; 3 Institute of Bioinformatics and Systems Biology, Helmholtz Zentrum München, Ingolstädter Landstraße 1, D-85764 Neuherberg, Germany; 4 Institute of Experimental Genetics, Genome Analysis Center, Helmholtz Zentrum München, Ingolstädter Landstraße 1, D-85764 Neuherberg, Germany; 5 Department of Medicine A, University Medicine Greifswald, Ferdinand-Sauerbruch-Str. NK, 17475 Greifswald, Germany; 6 Lehrstuhl für Experimentelle Genetik, Technische Universität München, 85350 Freising-Weihenstephan, Germany

**Keywords:** Inflammation, White blood cell count, C-reactive protein, Fibrinogen, Metabolomics, Mass spectrometry, Nuclear magnetic resonance spectroscopy

## Abstract

**Background:**

Inflammation occurs as an immediate protective response of the immune system to a harmful stimulus, whether locally confined or systemic. In contrast, a persisting, i.e., chronic, inflammatory state, even at a low-grade, is a well-known risk factor in the development of common diseases like diabetes or atherosclerosis. In clinical practice, laboratory markers like high-sensitivity C-reactive protein (hsCRP), white blood cell count (WBC), and fibrinogen, are used to reveal inflammatory processes. In order to gain a deeper insight regarding inflammation-related changes in metabolism, the present study assessed the metabolic patterns associated with alterations in inflammatory markers.

**Methods:**

Based on mass spectrometry and nuclear magnetic resonance spectroscopy we determined a comprehensive panel of 613 plasma and 587 urine metabolites among 925 apparently healthy individuals. Associations between inflammatory markers, namely hsCRP, WBC, and fibrinogen, and metabolite levels were tested by linear regression analyses controlling for common confounders. Additionally, we tested for a discriminative signature of an advanced inflammatory state using random forest analysis.

**Results:**

HsCRP, WBC, and fibrinogen were significantly associated with 71, 20, and 19 plasma and 22, 3, and 16 urine metabolites, respectively. Identified metabolites were related to the bradykinin system, involved in oxidative stress (e.g., glutamine or pipecolate) or linked to the urea cycle (e.g., ornithine or citrulline). In particular, urine 3’-sialyllactose was found as a novel metabolite related to inflammation. Prediction of an advanced inflammatory state based solely on 10 metabolites was well feasible (median AUC: 0.83).

**Conclusions:**

Comprehensive metabolic profiling confirmed the far-reaching impact of inflammatory processes on human metabolism. The identified metabolites included not only those already described as immune-modulatory but also completely novel patterns. Moreover, the observed alterations provide molecular links to inflammation-associated diseases like diabetes or cardiovascular disorders.

**Electronic supplementary material:**

The online version of this article (doi:10.1186/s12916-017-0974-6) contains supplementary material, which is available to authorized users.

## Background

Inflammation is a corporeal response to damaging stimuli associated with the activation of various molecular mechanisms. In addition to localized inflammatory reactions at the site of injury (redness, swelling, overheating, pain and disturbed function of the affected tissue or organ), reactions of the entire organism can be affected, depending on the severity of inflammation [1]. Both the local and systemic responses initiated by an inflammatory process indicate an imbalance in metabolism in the tissues affected. The metabolic disequilibrium at the site of injury is caused by the increased immune cell number (cells of the immune system flow through the increased blood flow to the injury site) and the diverse metabolic requirements of immune cells, which differ from those of local cells [2]. In addition to a general feeling of illness, there are a number of different inflammatory parameters that are of clinical importance, including high-sensitivity C-reactive protein (hsCRP), white blood cell count (WBC), and fibrinogen. Localized and systemic inflammatory reactions with changes in the classical inflammatory parameters are indications of the altered metabolism in the affected tissue [2]. After remission of the inflammatory response, tissue metabolism normalizes. If the remission process is interrupted, for example, due to persistence of pathogens, toxins, or other stimuli, damage of healthy tissue could occur. With respect to metabolic disease (e.g., diabetes), damage might be induced due to the adverse effect of over nutrition as the derived lipid species are able to induce the inflammatory response, i.e., induction of cytokine release, in resistant macrophages located in adipose tissue [3]. In general, chronic inflammation is considered integral to the development of serious systemic diseases such as type 2 diabetes mellitus (T2DM), cardiovascular diseases, gastrointestinal disorders, and rheumatoid arthritis.

CRP and fibrinogen are acute phase proteins [4] that are secreted from the liver upon interleukin 6 (IL-6) stimulation; IL-6 is released by activated macrophages and T cells. The primary role of CRP is binding to phospholipid species of damaged cells or pathogens to initiate the complement system. As the cause of tissue damage could be from various sources, elevations in hsCRP reflect a general inflammatory response rather than being attributed to a specific cause [5]. Another routinely measured indicator of systemic inflammation in particular is WBC [6]. Studies have shown elevated WBC levels not only during infection [7], but also in relation to cardiovascular diseases [8], T2DM [9], or metabolic syndrome [10]. Thus, all three inflammatory markers – hsCRP, fibrinogen, and WBC – are clinically useful in the diagnosis and follow-up of diseases.

Considering the close link of (chronic) inflammation with common diseases and its burden on the patient and the health system, improving the understanding about the metabolic implications of low-grade chronic inflammatory processes is an urgent need. A suitable tool for this purpose is metabolic profiling, as it allows the investigation of a broad range of small molecules (metabolites) in various body fluids. Consequently, the analysis of these metabolites in relation to inflammatory markers such as hsCRP, WBC, or fibrinogen enables the identification of alterations at the molecular level and thereby characterizes the (subclinical) inflammatory state. Previous studies have identified metabolites such as fatty acids, like arachidonic acid or palmitate, various lysophosphatidylcholines (lysoPC) (18:1 and 18:2), metabolites of purine metabolism, and amino acids associated with inflammatory processes in patients suffering from obesity [11], T2DM [12, 13], and their complications [14, 15].

To gain a deeper insight into inflammation-related metabolism, the present study combined non-targeted, i.e., based on liquid-chromatography coupled with tandem mass spectrometry (LC-MS/MS), and targeted, i.e., based on high-performance LC-MS/MS and ^1^H-NMR spectroscopy, metabolomics approaches. Through an investigation of metabolic patterns associated with alterations in hsCRP, WBC, and fibrinogen levels in humans in both plasma and urine, we are the first to generate a compilation of metabolites spanning multiple pathways, thus providing a link between metabolism and inflammation in a large non-diabetic sample of the general population.

## Methods

### Study population

The Study of Health in Pomerania (SHIP-TREND) is a population-based study located in West Pomerania, a rural region in north-east Germany [16]. A stratified (age, sex, and city/country of residence), random sample of 8826 adults aged 20–79 years was drawn from population registries. Sample selection was facilitated by centralization of local population registries in the Federal State of Mecklenburg, West Pomerania. Baseline examinations were conducted between 2008 and 2012. In total, 4420 subjects chose to participate (50.1% response). All participants gave written informed consent before taking part in the study. The study was approved by the ethics committee of the University of Greifswald and conformed to the principles of the Declaration of Helsinki. SHIP data are publicly available for scientific and quality control purposes. Data usage can be applied for via www.community-medicine.de.

Plasma and urine metabolomics data based on MS and NMR were available for a subsample of up to 1000 subjects without diabetes (self-reported). To address the aims of the present study we had to exclude subjects with missing values in inflammatory markers or confounding factors (*n* = 47), as well as those with acute inflammation defined as hsCRP > 10 mg/L (*n* = 28). In total, the present study comprised 925 subjects (Additional file 1: Figure S1).

### Laboratory measurements and phenotypic characterization

Smoking status (current, former or never smokers), daily alcohol consumption, and physical activity (≥1 h training a week) were assessed using computer-aided personal interviews. Waist circumference was measured to the nearest 0.1 cm using an inelastic tape midway between the lower rib margin and the iliac crest in the horizontal plane.

Fasting blood samples were taken from the cubital vein of participants in the supine position between 7.00 a.m. and 12.00 p.m. Over the same time span, spot urine samples were taken. Except for hsCRP and cystatin C, all clinical assays were performed using fresh plasma/serum samples. To this end, blood samples were immediately placed on ice. On average, 50 minutes elapsed between blood collection and measurement. Further, serum, EDTA-plasma, and urine samples were immediately partitioned into multiple aliquots and stored in a centralized biobank at –80 °C. Out of the frozen samples, metabolomics and hsCRP measurements were performed after completion of the study with an average storage time of 4.25 years. Fibrinogen concentrations were determined in citrate plasma samples according to Clauss, using a BCS-XP system (Siemens Healthcare Diagnostics, Eschborn, Germany). WBC concentration was determined in EDTA whole blood samples using the Sysmex XT 2000, XE 5000, or SE9000 analyzers (Sysmex, Kobe, Japan) or the Advia 2120i (Siemens Healthcare Diagnostics). Three levels of control material were measured by skilled technical personnel. Glucose, triglyceride, alanine aminotransferase, and hsCRP concentrations were measured using the Dimension Vista 500 analytical system (Dimension VISTA, Siemens Healthcare Diagnostics). Serum cystatin C concentrations were measured using a nephelometric assay (Dimension VISTA, Siemens Healthcare Diagnostics) with a functional sensitivity of 0.05 mg/L. Cystatin C-based estimated glomerular filtration rate (eGFR) was calculated using the CKD-EPI cystatin C equation: eGFR = 133 × min(serum cystatin C/0.8, 1)^-0.499^ × max(serum cystatin C/0.8, 1)^-1.328^ × 0.996^age^ [×0.932 if female] [17].

### Metabolomics measurements

A detailed description of all applied measurement techniques is provided in the Supplemental information. The following passage briefly summarizes the most important steps.

#### Metabolomics measurements based on MS

Non-targeted metabolomics analysis for metabolic profiling was conducted at the Genome Analysis Center, Helmholtz Zentrum München, Germany. Briefly, two separate LC-MS/MS analytical methods were used as previously published [18] to obtain broad metabolite spectra in plasma and urine samples in a non-targeted manner. After pre-processing, 475 plasma and 558 urine metabolites remained for the statistical analyses. Note that 177 plasma metabolites and 302 urine metabolites could not be unambiguously assigned to a chemical identity and are referred to hereafter with the notation “X” followed by a unique number.

Targeted metabolomics profiling of the plasma samples was performed using the AbsoluteIDQ p180 Kit (BIOCRATES LifeSciences AG, Innsbruck, Austria, online supplementary methods) and was conducted at the Institute of Clinical Chemistry and Laboratory Medicine, Universtiy Medicine Greifswald, Germany. This approach allows simultaneous absolute quantification of 188 metabolites using a combination of liquid chromatography (Agilent 1260 Infinity Binary LC, Santa Clara, United States) and mass spectrometry (AB SCIEX 5500 QTrap™ mass spectrometer; AB SCIEX, Darmstadt, Germany). After normalization and pre-processing of the data, 183 metabolites were available for subsequent analysis.

#### Metabolomics measurements based on ^1^H-NMR

Urine samples were measured on a Bruker DRX-400 NMR Spectrometer (Bruker BioSpin GmbH, Rheinstetten, Germany) operating at a ^1^H frequency of 400.13 MHz following a published workflow [19]. The Fourier-transformed and baseline-corrected NMR spectra were manually annotated by spectral pattern matching using Chenomx NMR Suite 6.1 (Chenomx Inc., Edmonton, Alberta, Canada) to deduce absolute urinary concentrations of 51 metabolites.

#### Data integration

Most of the metabolites were unique to one of the applied techniques. However, 44 and 22 metabolites in plasma and urine, respectively, were overlapping with at least one other technique. With respect to plasma, following the grouping of metabolites in biochemical classes (i.e., lipids, amino acids and carbohydrates), correlations of those metabolites measured on both platforms were computed with all members of the same biochemical class. Subsequently, the metabolite with the higher median correlation across all class members was kept for further analysis. Regarding the lower sensitivity of NMR compared to MS, in cases of duplicated measures, those obtained on the MS were kept for further analyses. In total, 613 plasma and 587 urine metabolites were used in the subsequent statistical analyses.

### Statistical analysis

Continuous data are expressed as median (25th–75th quartile) and nominal data are expressed as percentage. For bivariate analyses, the Mann–Whitney U-test (continuous data) or χ^2^ test (nominal data) were used to compare women and men. Linear regression models were performed to assess the association between inflammatory markers, namely, hsCRP, WBC, and fibrinogen (independent), and plasma and urine metabolites (dependent). Concentrations of all metabolites as well as hsCRP levels were log-transformed to fulfil the requirements of linear regression. Models were adjusted for age, sex, waist circumference, smoking behavior and physical activity. To avoid spurious results in linear regression analysis, univariate outliers for each metabolite were excluded whenever concentrations exceeded more than three standard deviations from the mean value. Sensitivity analyses on the influence of latent diabetic subjects (*n* = 29; meeting one of the following criteria: (1) fasting glucose > 11.1 nmol/L, or (2) HbA1c > 6.5%, or (3) intake of diabetic medication (ATC code A10)) was performed by exclusion of those subjects. To account for multiple testing, we adjusted the *P* values from regression analyses by controlling the false discovery rate (FDR) at 5% using the Benjamini–Hochberg procedure.

Classification analyses were performed to prove the discriminative ability of the metabolome as well as to highlight central metabolites. As no established cut-offs for low-grade inflammation exist we defined an advanced inflammatory state using the population distributions of the inflammatory markers, i.e., participants with hsCRP, WBC, and fibrinogen levels, by denoting all of those in the upper quartile as having an advanced inflammatory state. In contrast, a healthy state was defined as being consistently in the lower quartile of inflammatory markers. As a result, 42 and 54 subjects presented with low or high inflammatory conditions, respectively. For classification purposes, a random forest was applied embedded in a two-stage cross-validation procedure (Additional file 1: Figure S2). The random forest was implemented in R via the *randomForest* package (v 4.6-10) [20]. This combination allowed for variable selection and validation of a defined subset (limited to 10 metabolites), even in the absence of an independent test set. Further, evaluation of multiple permutations among training, testing, and validation sets allowed us (1) to achieve a reliable estimate of the discriminative performance of the metabolome and (2) to judge the consistency of the named markers to discriminate an inflammatory state. For this purpose, 872 metabolites with less than 20% missing values were chosen along with participant age, sex, waist circumference, and eGFR as additional variables. Missing values were imputed using the k-nearest neighbor technique as implemented in the R package *impute*, setting k to 10.

Statistical analyses were performed using SAS version 9.4 (SAS statistical software, version 9.4, SAS Institute, Inc., NC, USA) and R 3.1.1 (R Foundation for statistical computing, version 3.1.1, Vienna, Austria).

## Results

The general characteristics of the study population are provided in Table 1. Men and women were 50 years old on average. Men were less often never smokers, had a higher waist circumference, and exhibited higher levels of glucose, triglycerides, and eGFR, whereas cholesterol levels were slightly lower compared to those of women. With respect to inflammatory markers, no differences were detected for WBC, whereas fibrinogen and hsCRP were lower in men than in women.

**Table 1:** General characteristics of the study population

	Men (n = 415)	Women (n = 510)	*P*
Age, years	50 (40–61)	51 (41–60)	0.86
Smoking, %			<0.01
Never smoker	30.8	50.6	
Ex-smoker	46.0	29.2	
Current smoker	23.1	20.2	
Physical activity (≥1 h per week), %	72.8	73.5	0.82
Waist circumference, cm	94 (87–102)	81 (74–90)	<0.01
Glucose, mmol/L	5.4 (5.1–5.8)	5.2 (4.9–5.6)	<0.01
Triglycerides, mmol/L	1.32 (0.93–1.93)	1.16 (0.85–1.61)	<0.01
Cholesterol, mmol/L	5.4 (4.6–6.1)	5.5 (4.9–6.3)	<0.01
eGFR, mL/min/1.72 m^2^	117 (108–125)	112 (103–121)	<0.01
WBC, Gpt/L	5.34 (4.64–6.35)	5.50 (4.78–6.47)	0.18
Fibrinogen, g/L	2.80 (2.30–3.30)	3.10 (2.60–3.50)	<0.01
hsCRP, mg/L	1.00 (0.55–1.83)	1.26 (0.67–2.83)	<0.01

Continuous data are expressed as median (25th percentile–75th percentile); nominal data are given as percentages

*χ^2^ (nominal data) or Mann–Whitney (interval data) tests were performed

*eGFR* estimated glomerular filtration rate, *WBC* white blood cell count, *hsCRP* high-sensitive C-reactive protein

### Associations with inflammatory markers in the plasma metabolome

**Fig. 1:**
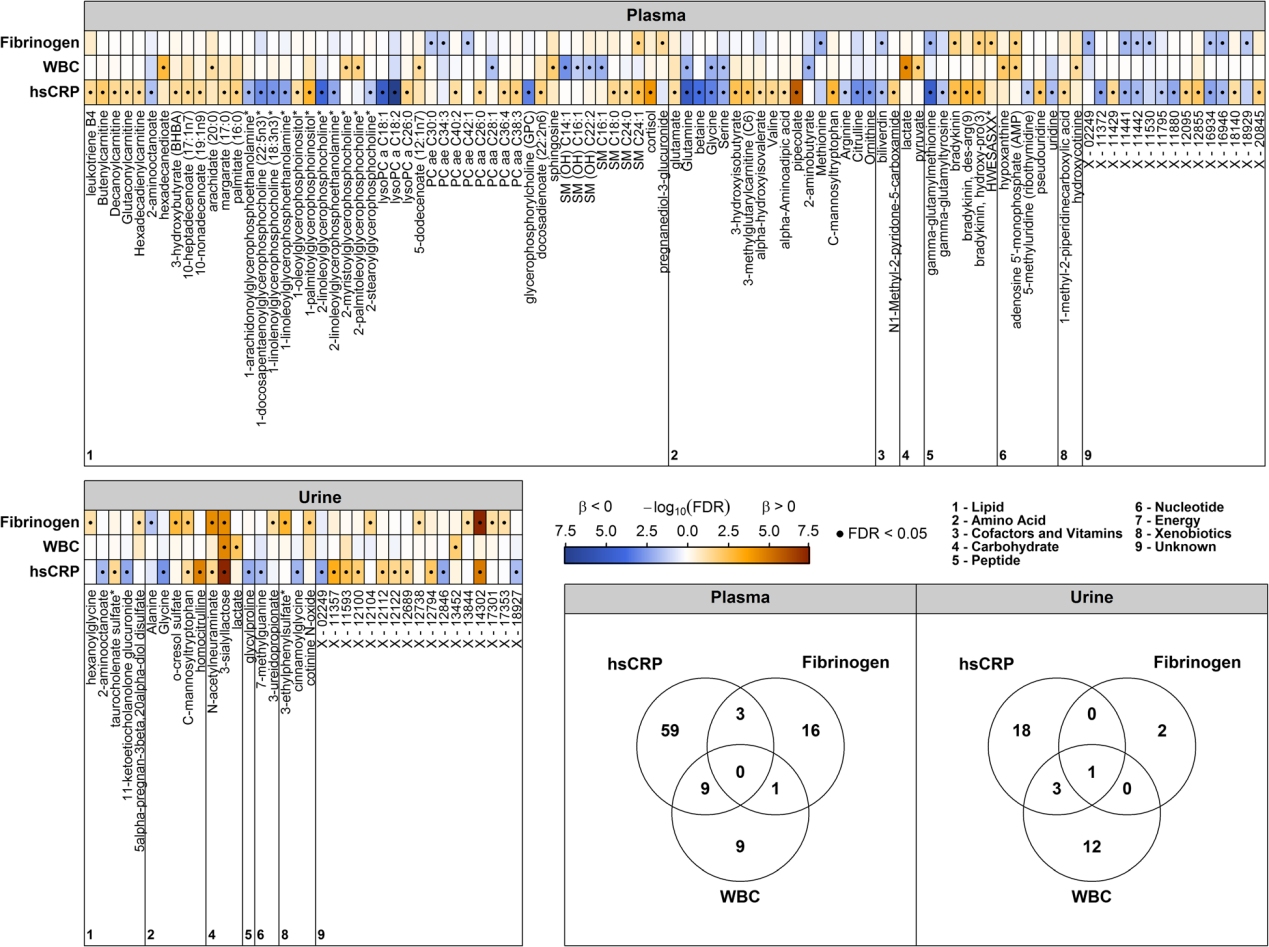
Color coded corrected *P* values (controlling the false discovery rate (FDR) at 0.05) from linear regression analyses for the association of high-sensitivity C-reactive protein (hsCRP), white blood cell count (WBC), or fibrinogen with plasma (*upper panel*) or urine metabolites (*lower left panel*). Significant associations (FDR < 0.05) are marked with a dot. All analyses were adjusted for age, sex, waist circumference, smoking behavior, and physical activity. Orange and blue shading indicate positive and negative associations, respectively. Corresponding beta estimates and FDR values are given in Additional file 2: Tables S1 and S2. *Lower right panel*: Venn diagrams depict the overlap in significantly associated metabolites to each trait in plasma and urine

**Fig. 2:**
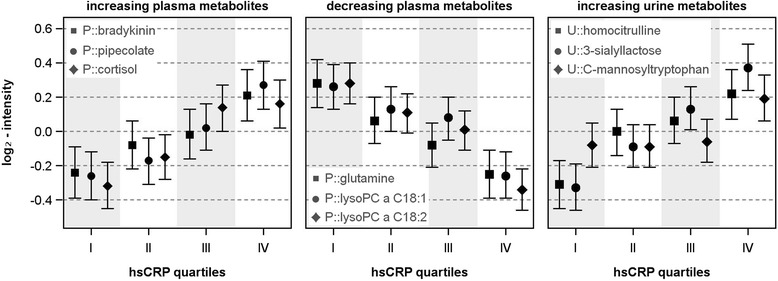
Predicted least square means with 95% confidence intervals from linear regression analyses using quartiles of high-sensitivity C-reactive protein (hsCRP) as exposure controlling for age, sex, waist circumference, physical activity and smoking

HsCRP, WBC, and fibrinogen showed significant associations with 71, 20, and 19 out of 613 plasma metabolites, respectively; none was common to all traits (Fig. 1, Additional file 2: Table S1). Bradykinin metabolites, biliverdin, gamma-glutamylmethionine, the sphingomyelin C24:1, the amino acids glutamine, glycine and serine, and some compounds with unknown chemical identity were shared by at least two of the assessed traits. A wealth of unique associations with hsCRP became apparent, including positive associations with intermediates of branched-chain amino acid and lysine degradation (e.g., valine or pipecolate (Figs. 1 and 2)), acylcarnitines, long-chain fatty acids, further sphingomyelins, and diacylphosphatidylcholines, as well as cortisol levels (Fig. 2). Significant inverse associations with hsCRP became apparent with respect to members of the urea cycle and several lysophosphatidylcholines (lysoPC) containing (poly)unsaturated fatty acids (e.g., lysoPC 18:1 and 18:2; Fig. 2). The most prominent findings unique to WBC were the positive associations with lactate, pyruvate, sphingosine, and hexadecanedioate. Unique associations with fibrinogen were observed for plasma levels of pregnanediol-3-glucuronide (positively) and phosphatidylcholines with fatty acid residues bound to one acyl and one alkyl group (inversely).

### Associations with inflammatory markers in the urine metabolome

In general, associations with urinary metabolites were less frequent compared with plasma metabolites (Fig. 1 and Additional file 2: Table S2); HsCRP, WBC, and fibrinogen showed significant associations with 22, 3, and 16 out of 587 urine metabolites, respectively (Fig. 1). A positive association with the disaccharide 3’-sialyllactose was common to all investigated traits; further, it was the strongest association within the present study (Fig. 1 and Additional file 2: Table S2). The related metabolite N-acetylneuraminate (sialic acid) was positively associated with hsCRP and fibrinogen similarly to C-mannosyltryptophan (Fig. 2) and the unknown X-14302. Similar to plasma, hsCRP comprised most of the unique associations (Fig. 1), including homocitrulline, taurocholate sulfate and several unknown compounds (positively) as well as glycine, 2-aminooctanoate, glycylproline, and 7-methylguanine (inversely). Unique associations with fibrinogen consisted of 3-ureidopropionate, hexanoylglycine, 5-alpha-pregnan-3-beta,20-alpha-diol disulfate, o-cresol sulfate, and the xenobiotics 3-ethylphenylsulfate and cotinine N-oxide.

The vast majority of the presented results remained stable after exclusion of 29 latent diabetic subjects (Additional file 1: Figure S3). Single departures might be most likely due to random variation as comparison between the false discovery rate values of the whole and the subpopulation revealed no gross effects (Additional file 1: Figure S3).

### Predicting an advanced inflammatory state

**Fig. 3:**
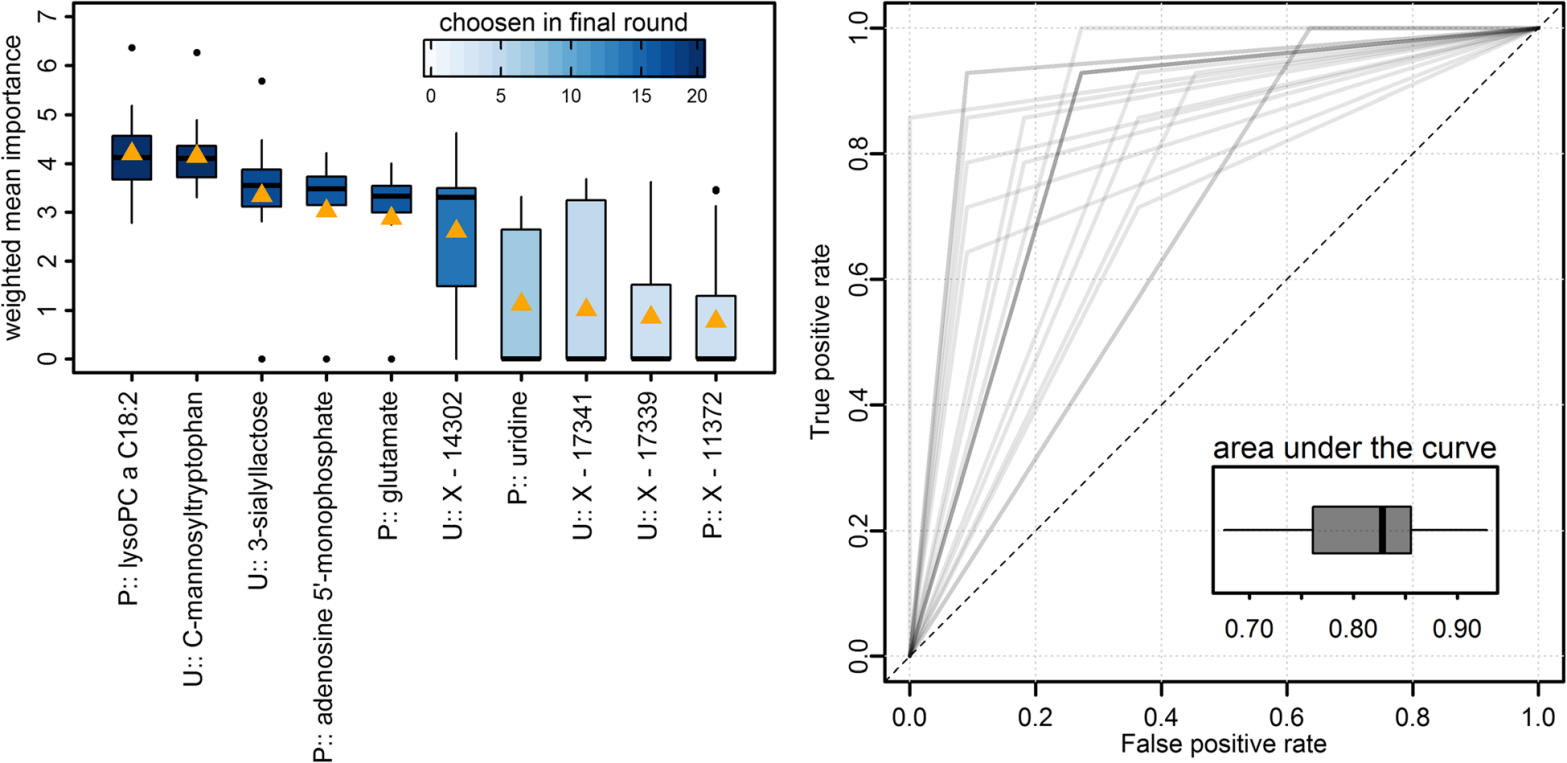
Final results from classification analyses using random forests in a two-stage cross-validation scheme with 20 inner and 20 outer loops (Additional file 1: Figure S1). *Left panel*: Ten most important metabolites ranked by a weighted (area under the curve) mean Gini index (orange triangle). Boxplots indicate distribution across 20 outer runs. *Right panel*: Receiver operating characteristic (ROC) curve and boxplot of the area under the curve from 20 outer loops. Overlapping ROCs are displayed by darker shades

In addition to association testing, we searched for a discriminative signature between healthy participants (only low inflammatory markers) and an advanced inflammatory state, defined as the consistent presence of inflammatory markers in the highest quartile among the present cohort (*n* = 54). To this end, we applied a random forest analysis embedded in a two-stage cross-validation procedure combining variable selection with model evaluation. We achieved moderately robust classification as shown by a median area under the receiver operating characteristic curve of 0.83 across 20 validation runs using a subset of 10 metabolites (Fig. 3). The approach clearly highlights the importance of five metabolites, consisting of three plasma metabolites, lysoPC 18:2, adenosine 5’-phosphate, and glutamate, as well as two urine metabolites, C-mannosyltryptophan and 3’-sialyllactose.

## Discussion

Chronic low-grade inflammation as reflected by elevations in blood parameters, such as hsCRP, WBC, or fibrinogen, is accompanied by a multitude of metabolic changes. In an attempt to comprehensively characterize such changes, the present study used a state-of-the-art metabolomics approach comprising non-targeted and targeted techniques in an apparently healthy sample of the general population. The absence of acute inflammatory processes as well as diabetic cases allowed the profiling of subclinical molecular patterns. This profiling provided novel insights in altered pathways possibly contributing to the adverse effect of inflammatory processes on metabolic and cardiovascular diseases. Urinary metabolites in particular might be promising biomarkers to ease the diagnosis and monitoring of inflammation-associated diseases.

### The bradykinin systems as proof of principle

One common feature of hsCRP and fibrinogen indicating low-grade inflammation was the increased appearance of bradykinin metabolites in plasma. Bradykinin, as part of the kinin-kallikrein system, is released from high-molecular-weight kininogen upon interaction with kallikrein, which in turn is activated by factor XIIa in the course of activated coagulation [21]. Once released, bradykinin acts as a vasoactive peptide hormone via binding to bradykinin receptors, B1 and B2, in the vascular endothelium and induces the classical symptoms of inflammation, i.e., swelling and redness [21]. However, increasing vascular permeability and enhancement of smooth muscle tone are only two of several mechanisms of bradykinin action [22]. Bradykinin plays a particularly important role in various classifications of angioedema, but also in other inflammatory processes such as allergic rhinitis [21]. Conflicting reports exist about its relation with the cardiovascular system. Moreover, bradykinin production leads to the release of various cytokines [23], e.g., IL-6, which further activates the innate immune system and, thereby, CRP release from the liver. In this respect, our findings mirror previously described biological pathways emphasizing the validity of the presented approach.

### Metabolites linked to an oxidative stress signature

The strongest associations with respect to amino acids were demonstrated for plasma levels of glutamine (negatively) and pipecolate (positively) (Fig. 2). Pipecolate, a metabolite in the degradation of the essential amino acid lysine, has shown antioxidative properties in a number of cell and animal models [24]. A possible explanation for its association with hsCRP might be an increased demand of antioxidant agents during inflammation in tissues highly active in respiration. One opportunity to compensate the increased energy demand accompanied by inflammatory processes would be an increased utilization of fat from deposits, such as white adipose tissue. Indeed, within the present study, an increase in hsCRP was associated with increased plasma levels of free fatty acids. After uptake in respiratory active tissues, free fatty acids are subjected to oxidation within the mitochondria. To pass the outer mitochondrial membrane, the fatty acids have to be conjugated with carnitine, yielding acylcarnitines. In situations of pronounced lipolysis and respiration, these intermediates are likely to be spilled into the circulation, which is further supported by our observations. A possible mediator in this context might be the elevated cortisol concentrations observed in the present study. Cortisol is well-known to counteract a prolonged inflammatory milieu by diminishing the production of pro-inflammatory mediators, including IL-12, IFN-γ, or TNF-α, at the transcriptional level. Importantly, the interrelation between cortisol and cytokines is mutual. An acute injection of cytokines led to a rise in cortisol levels, seen as an adaptive response to fulfil the increased energy demand of inflamed tissues [25]. Consistently, an experimental study in healthy volunteers [26] showed a cortisol-dependent induction of lipolysis from femoral and abdominal adipose tissue.

It should be noted that even microbiota are able to produce pipecolate [27] when given lysine as a substrate, and host dysbiosis is a hallmark of many inflammatory diseases [28, 29]. As a direct link between plasma pipecolate levels and composition of the gut microbiota has not been available up to now, further studies are needed to investigate this possible line of explanation in more detail.

In contrast to the positive association with pipecolate, we observed a significant inverse association of hsCRP with glutamine and to a lesser extent with WBC. The non-essential amino acid glutamine attenuated inflammatory signs when given as a supplement in clinical applications for patient recovery after abdominal surgery [30] and in the perioperative period [31, 32], as well as in patients who needed intensive care. In addition to the lowering of inflammatory markers, e.g., hsCRP, TNF-α, or IL-6, even surrogates of intestinal permeability were reduced [30]. The authors concluded that glutamine is able to effectively reduce inflammation. This finding is in concordance with the inverse association between hsCRP and glutamine in the present study. It is known that glutamine, as a precursor of glutathione, plays a crucial role in the prevention of oxidative damage in the human body [33] and thus also has a positive influence on the maintenance of the gastrointestinal barrier [30] and the endothelial function of the blood vessels [34]. An intervention study among women of advanced age showed that a 6-month administration of amino acids, including glutamine, significantly reduced hsCRP and thus has potential beneficial effects [34].

### Inverse association between inflammation and members of the urea cycle

In addition to its role as precursor of glutathione, glutamine is implicated in arginine metabolism. Arginine is an amino acid of the urea cycle [35] and was inversely associated with hsCRP in the present study. Previous investigations have suggested an immune-modulating effect [36] and anti-inflammatory properties [37] of arginine. Alterations in the urea cycle with respect to hsCRP were further highlighted by inverse associations with the plasma metabolites ornithine and citrulline as well as a strong association with urinary homocitrulline. The latter is detected in large quantities in urine in cases of urea cycle disorders [38]. These findings might be a general feature of (severe) inflammatory conditions, as a previous study observed decreased levels of urea cycle intermediates in subjects with sepsis and trauma [39]. Interestingly, during the recovery phase and in the reduction of inflammation, a rise in arginine and citrulline levels were observed. Furthermore, among critically ill patients, low plasma citrulline levels were related to low levels of arginine and glutamine as well as elevated hsCRP levels [40]. Even if the underlying mechanisms are not yet understood in detail, it can be assumed that glutamine, with its central position in metabolism, plays a crucial role in inflammation. However, longitudinal studies are needed to assess the possible anti-inflammatory effect of high baseline glutamine concentrations.

### Phospholipids as potential inflammatory mediators

A very prominent molecular signature associated with hsCRP consisted of lysoglycerophospholipds with choline (lysoPC), inositol, or ethanolamine as the head-group and unsaturated fatty acids as residues merely in an inverse manner. Lysoglycerophospholipds are derived from hydrolysis of phospholipids and this might point towards diminished phospholipase A1 or A2 activity. In contrast to our findings, several lines of evidence suggest increased phospholipase activity under inflammatory conditions [41–43]. A diminished production could therefore be reasonable, as one of the primary precursors of lysoPC, glycerophosphorylcholine, also decreased with increasing hsCRP levels. LysoPCs have often been discussed as pro-inflammatory molecules with regard to acute and chronic inflammation and atherosclerosis [44] and are strongly suspected to be associated with inflammatory diseases, e.g., rheumatoid arthritis [45]. LysoPCs transport fatty acids, phosphatidylglycerol, and cholines between different tissues, but also activate signaling pathways leading to the release of second messengers [44] as ligands for G protein-coupled receptors [44]. Thus, they are able to promote the release of pro-inflammatory factors in endothelial cells – an important mechanism in the development of arteriosclerosis [46]. Interestingly, population-based studies revealed inverse associations between lysoPC 18:1 and 18:2 with the risk of developing T2DM [47] or the onset of coronary heart disease [48]. Given that a chronic inflammatory environment is a hallmark of such diseases, our observation of an inverse association with hsCRP potentially further argues for a functional implication of lysoPCs in the mediation between inflammation and associated diseases. The latter might be further supported by the missing effect of the exclusion of latent diabetic subjects on the observed associations between lysoPC 18:1 and 18:2 and hsCRP levels.

### 3’-sialyllactose as a novel link to inflammation

The urinary metabolite 3’-sialyllactose showed the strongest association and was positively related to all investigated traits. 3'-sialyllactose belongs to the group of oligosaccharides, which are generally components of milk and have a protective, immune-modulating influence on the development of neonates [49, 50]; 3'-sialyllactose is composed of sialic acid (N-acetylneuraminic acid) and lactose [51]. Interestingly, even urinary sialic acid levels rose under inflammatory conditions, particularly in respect to fibrinogen levels but also to hsCRP within the present study. Sialic acid forms the outer end of the sugar branches representing the glycocalyx [52, 53] and exerts influence on immune reactions in different ways [53]. Briefly, sialic acid takes part in the development of B and T cells [54, 55] and plays a role in the interaction of immune cells with other cells [56]. The diapedesis of leucocytes [57] is mediated by cellular adhesion molecules with selectins and sialic acid representing important components of ligands, which can bind to the selectin family of the cell adhesion molecules. A positive relation between sialic acid and hsCRP was reported among 48 patients with chronic cardiac insufficiency, and the authors postulated both as reliable markers of systemic inflammation in chronic heart failure patients [58]. Moreover, sialic acid appears to be a pro-inflammatory marker not only in the case of chronic cardiac insufficiency, but also in other cardiovascular diseases such as deep venous thrombosis [59], T2DM [60], and the T2DM-associated sequelae diabetic nephropathy [61]. These findings are in good agreement with the positive association of sialic acid or 3'-sialyllactose and hsCRP in our study. As already mentioned with respect to the lysoPCs, even the association with 3-siallylactose was not affected by the exclusion of latent diabetic subjects and thus might point towards elevated 3-siallylactose levels as a preceding event. More astonishing is the discrepancy with the above-mentioned protective, immune-modulating effects of 3'-sialyllactose in neonates [49, 50]. It would be conceivable that the immune-promoting, anti-inflammatory effects of 3'-sialyllactose are only effective in newborns whose immune systems are still developing, whereas in adults, apparently the exact opposite effect is achieved. This situation should be further investigated through intervention studies.

### Similarities and difference between inflammatory surrogates

The strong differences observed in the metabolic signatures for each of the inflammatory traits must be discussed. All markers were of different specificity for the inflammatory process and, from a statistical point of view, were only moderately intercorrelated (Additional file 1: Figure S3). With respect to an acute inflammatory response, CRP, fibrinogen, and WBC belong to different stages of the acute phase response with different temporal patters of accumulation [62, 63]. However, the present study focused on a low-grade inflammatory state characterized by mildly elevated hsCRP, WBC, or fibrinogen levels. The difference between hsCRP and WBC may be due to their different origins and roles during a chronic inflammatory response. CRP (and fibrinogen) are produced and secreted by the liver upon IL-6 stimulation and subsequently reach the site of inflammation through circulation. Even leucocytes reach the inflammatory site through circulation following release from the bone marrow. However, the inflammatory state of, for example, adipose tissue, in the course of low-grade inflammation is not primarily due to newly arriving immune cells but rather due to the polarization of resistant macrophages secreting cytokines, such as IL-6, which in turn stimulate the liver to secrete, among others, CRP. In conclusion, the differing metabolic profiles observed reflect the distinct physiological roles of the parameters. Even though the latter might be obvious, our results further argue for a distinct discussion of a low-grade inflammatory state dependent on the markers used to define it. The prominent role of CRP might thereby most likely be due to its long half-life, the highly sensitive assay applied, and its non-specific nature reflecting multiple distinct processes.

With respect to the different association profiles of all considered markers the unique positive relation between WBC and the carbohydrates lactate and pyruvate might be of particular interest. Lactate is known to accumulate at sites of inflammation [2], not only due to hypoxic conditions forcing the switch to aerobic glycolysis, but also to meet the general demands of the highly proliferative immune cells. Moreover, lactate itself was recently shown to modulate the pro-inflammatory response of T cells [64]. Importantly, lactate induced the trapping of T cells at the site of inflammation and thus the persistence of pro-inflammatory signaling.

### A predictive metabolic signature

Random forest analyses revealed a good capacity of the metabolome to predict an advanced inflammatory state. Within this signature, four metabolites were clearly distinguished as they were robustly included in the final classifier independently of the subpopulation used. The possible relation between lysoPC 18:1 and urinary 3’-sialylactose has already been discussed in the previous sections. C-mannosyltryptophan is a product derived from the C-glycosylation of the aromatic amino acid tryptophan and mannose. Previous studies noted a strong age-dependency of plasma C-mannosyltryptophan [65, 66], likely due to its strong dependency on renal filtration [67, 68]. However, neither age nor eGFR emerged as comparable predictive variables in the classification procedure. In the context of inflammation, it might be of interest that C-glycosylated tryptophan can also be found in IL-12 and components of the complement system [69]. However, all of these studies refer to plasma levels of C-mannosyltrytophan and, even if these were also associated with hsCRP within the present study, the possible interplay of kidney function and inflammation warrants further investigation given the pronounced association observed in urine.

### A note on the applicability of our findings

The comprehensive metabolic picture of low-grade inflammation drawn in the present study is useful for different kinds of research. Our results add a subclinical inflammatory state as an important determinant of the metabolome, which has to be considered in future metabolomics studies not only to avoid artificial findings but also to interpret metabolic patterns observed. The latter is of particular interest as metabolomics is a rather young scientific branch and the knowledge about its modulators is still incomplete. Further, key metabolites discussed and presented here are valuable targets for future studies to improve the diagnosis and treatment of inflammatory-associated diseases. Moreover, those metabolites might further provide molecular links between a low-grade inflammatory state and metabolic disease as small molecules are increasingly recognized as mediators in disease progression [70].

### Strengths and limitations

An undeniable strength of our study is the combination of a large sample size, the comprehensive profiling of the metabolome, and the measurement of three clinically important inflammatory parameters. All participants were apparently healthy with respect to acute inflammation. Thus, we were able to (1) confirm previous findings concerning known associations, (2) gain deeper insights in the broad field of metabolomics, and (3) give recommendations for new possible markers indicating low-grade inflammation. However, it must be mentioned that, due to the cross-sectional design of our investigation, no conclusions can be drawn about the causalities of the found associations. Intervention studies are necessary to investigate the metabolic fingerprint of inflammation causally. Additional studies should be performed profiling the metabolic signatures of other established inflammatory markers, such as IL-6 or TNF-α, in order to augment the acquired findings, in particular with respect to the remission of inflammation. As our study addressed a low-grade inflammatory state in particular, further studies in cohorts of patients suffering from inflammatory disease, e.g., arthritis or chronic pancreatitis, are required to judge the clinical usefulness of the presented metabolic markers of inflammation. With respect to possible bystander effects, we could not rule out hidden mediation not captured by the data obtained for the study population.

## Conclusions

We were the first to investigate the metabolic profile of an apparently healthy sample of the general population regarding its association with three clinically important representatives of inflammatory parameters. We observed an extensive picture of various associated plasma and urine metabolites, among them classical representatives of inflammation, e.g., the bradykinin system. Importantly, we were able to describe novel metabolites that were not yet investigated with regards to their connection with inflammation such as 3’-sialyllactose; these may prove potential, novel non-invasive biomarkers for low-grade inflammatory processes. As the link between metabolism and inflammation is an understudied phenomenon [11], our study suggests promising biomarkers for the actions of hsCRP, WBC, and fibrinogen on metabolism, which promise potential support in diagnosis and prognosis. Further, a preventive aspect might be of interest, for example, as shown for the inverse relation between glutamine and hsCRP, which was attenuated by glutamine supplementation. Similar strategies may be possible for other central metabolites named in the present study.

## Additional files

Additional file 1: Supplemental Matrial, Methods and Figures. (DOCX 678 kb)

Additional file 2: Supplemental Tables. (XLSX 30 kb)

## Abbreviations

eGFR: estimated glomerular filtration rate
hsCRP: high-sensitivity C-reactive protein
IL: interleukin
lysoPC: lysophosphatidylcholine
MS: mass spectrometry
NMR: nuclear magnetic resonance spectroscopy
SHIP: Study of Health in Pomerania
T2DM: type 2 diabetes mellitus
WBC: white blood cell count
