# Supplementary material for: Comprehensive metabolic profiling of chronic low-grade inflammation among generally healthy individuals
Source: BMC Med. 2017 Nov 30;15:210. doi: 10.1186/s12916-017-0974-6 (PMC5708081; doi:10.1186/s12916-017-0974-6)
Supplement: Supplementary file 1 — Supplemental Matrial, Methods and Figures. (DOCX 678 kb) [file 12916_2017_974_MOESM1_ESM.docx]

**SUPPLEMENTAL INFORMATION**

**Comprehensive metabolic profiling of chronic low-grade inflammation among general healthy individuals**

**METHODS**

***Untargeted LC-MS/MS profiling***

*Metabolomics Measurements*

Non-targeted metabolomics analysis for metabolic profiling was conducted at the Genome Analysis Center, Helmholtz Zentrum München. Two separate LC-MS/MS analytical methods mere used as previously published, i.e. in positive and in negative ionization modes, were used to detect a broad metabolite panel [1]. In this study, samples were divided into two sets according to the biological matrices of the samples, i.e. plasma and urine. On the day of extraction, samples were thawed on ice. A 100µL of the sample were pipetted into a 2mL 96-well plate. In addition to study samples, a human pooled reference plasma sample (Seralab, West Sussex, United Kingdom) and another pooled reference matrix of each sample set (Seralab, West Sussex, United Kingdom) were extracted and placed in 1 and 6 wells, respectively, of the 96-well plate. These samples served as technical replicates throughout the data set to assess process variability. Beside those samples, 100μL of water was extracted as samples and placed in 6 wells of the 96-well plate to serve as process blanks. Protein was precipitated and the metabolites were extracted with 475µL methanol, containing four recovery standards to monitor the extraction efficiency. After centrifugation, the supernatant was split into 4 aliquots of 100µL each onto two 96-well microplates. The first 2 aliquots were used for LC-MS/MS analysis in positive and negative electrospray ionization mode. Two further aliquots were kept as a reserve. The extracts were dried on a TurboVap 96 (Zymark, Sotax, Lörrach, Germany). Prior to LC-MS/MS in positive ion mode, the samples were reconstituted with 0.1% formic acid (50µl for plasma, 100µl for urine). Whereas samples analyzed in negative ion mode were reconstituted with 6.5mM ammonium bicarbonate (50µl for plasma, 100µl for urine), pH 8.0. Reconstitution solvents for both ionization modes contained internal standards that allowed monitoring of instrument performance and also served as retention reference markers. To minimize human error, liquid handling was performed on a Hamilton Microlab STAR robot (Hamilton Bonaduz AG, Bonaduz, Switzerland). LC-MS/MS analysis was performed on a linear ion trap LTQ XL mass spectrometer (Thermo Fisher Scientific GmbH, Dreieich, Germany) coupled with a Waters Acquity UPLC system (Waters GmbH, Eschborn, Germany). Two separate columns (2.1 x 100 mm Waters BEH C18, 1.7 µm particle-size) were used either for acidic (solvent A: 0.1% formic acid in water, solvent B: 0.1% formic acid in methanol) and or for basic (A: 6.5mM ammonium bicarbonate, pH 8.0, B: 6.5mM ammonium bicarbonate in 95% methanol) mobile phase conditions, optimized for positive and negative electrospray ionization, respectively. After injection of the sample extracts, the columns were developed in a gradient of 99.5% A to 98% B over an 11 min run time at 350µLl/min flow rate. The eluent flow was directly run through the ESI source of the LTQ XL mass spectrometer. The mass spectrometer analysis alternated between MS and data-dependent MS/MS scans using dynamic exclusion and the scan range was from 80-1000 m/z. Metabolites were identified by Metabolon, Inc. from the LC-MS/MS data by automated multiparametric comparison with a proprietary library, containing retention times, m/z ratios, and related adduct/ fragment spectra[2]. Identification criteria for the detected metabolites are described in Evans *et al.*[1]. Quality control methods and normalization of metabolite levels are explained in detail in the supplement.

*Metabolomics Measurements: Quality Control and Normalization of Metabolite Levels*

To correct for daily variations of platform performance, the raw ion count of each metabolite was rescaled by the respective median value of the run day. Valid estimation of the median was ensured by keeping only metabolites with at least three measured values on more than the half of the run days. This procedure resulted in 475 and 558 metabolites for plasma and urine, respectively, available for the present analysis. 263 metabolites were measured in both bio fluids. We chose probabilistic quotient normalization (PQN) [3] to account for diurnal variation of urine samples, since this procedure was shown to be superior to the common creatinine scaling. For this purpose we calculated a mean-pseudo-spectrum depending on metabolites with measurements for all participants (131 urine metabolites). Subsequently, we calculated a dilution factor as the median quotient between the reference spectrum and each sample. Of note, urine creatinine and the estimated dilution factor were highly correlated (r=0.91, p<0.001) within the present study sample. Afterwards all metabolite levels were log_2_-transformed. Separately for plasma and urine samples we performed multivariate outlier detection using an algorithm proposed by Filzmoser *et al.* [4] as implemented in the *pcout* function within the R package *mvoutlier*. The algorithm provides an outlier score for each sample based on a weighted combination of location and scatter estimations using principle component analysis and the Mahalanobis distance on a robustly scaled data matrix. The default parameters were used for the identification process, except the critical value for the location outliers was set to 4, as it corresponds to a 4 SD exclusion criteria. The minimum score was used as cut-off for outlier identification. As a result 13 and 8 samples from plasma and urine were excluded, respectively.

***Targeted LC-MS/MS profiling***

*Metabolomics Measurements*

Targeted metabolomics profiling of the serum samples was performed using the AbsoluteIDQ p180 Kit (BIOCRATES LifeSciences AG, Innsbruck, Austria, online supplementary methods). 10 µl aliquots of each plasma sample were processed as recommended by the manufacturer. The fully automated assay combined flow injection (FIA) and LC-MS/MS selective detection using MRM pairs and quantifies up to 188 metabolites from 5 different compound classes. Via FIA acyl carnitines, phospho- and sphingolipids were measured in positive ionization mode and the sum of hexoses in negative ionization mode. With a LC-MS/MS analytical method, under the use of an Agilent C18 column, amino acids and biogenic amines were detected. MS analyses were performed on an AB SCIEX 5500 QTrap™ mass spectrometer (AB SCIEX, Darmstadt, Germany) with electrospray ionization combined with a HPLC system (Agilent 1260 Infinity Binary LC, Santa Clara, United States) including a degasser unit, column oven, autosampler and a binary pump. Internal standards (isotope labelled) are partially integrated in the Kit plate for metabolite quantification. After the measurement a pre-processing step, includes peak integration and concentration determination from calibration curves, with Analyst software (Version 1.5.1, AB Sciex, Darmstadt, Germany), data were uploaded into Biocrates MetIDQ software (part of the kit) and the metabolite concentrations were automatically calculated with it.

*Metabolomics Measurements: Quality Control and Normalization of Metabolite Levels*

To account for between plate variation, a solely sample dependent normalization was performed. To this end for each plate the measured concentrations of the metabolites were divided by the median concentration leading to equal median values for each metabolite on each plate. Subsequently, the median of the plate medians was calculated to reset to the original scale (µM concentrations). No obvious pattern in missing values along the measurement period became obvious. However, only metabolites with at least 20% valid observations were included in the final data sets, resulting in 183 used for subsequent analysis. PCA was performed to detect multivariate outliers. These were defined as samples deviating more than three times the standard deviation (SD) from the mean Mahalanobis distance based on the first ten principle components. As a result, four samples were excluded. Finally, metabolite levels were log_2_-transformed.

***^1^H-NMR profiling***

*Metabolomics Measurements*

Prior analysis urine specimens were stored about five years at -80°C. After thawing, urine specimens were centrifuged for 5 min at 3000g and the supernatant was used for spectroscopic analysis. To this purpose, 450 µl urine were mixed with 50 µl phosphate buffer in order to stabilize the urinary pH at 7.0 (±0.35). The phosphate buffer was prepared with D2O and contained sodium 3-trimethylsilyl-(2,2,3,3-D4)-1-propionate (TSP) as reference. Spectra were recorded at the University Medicine Greifswald, Germany, on a Bruker DRX-400 NMR spectrometer (Bruker BioSpin GmbH, Rheinstetten, Germany) operating at 1H frequency of 400.13 MHz and equipped with a 4-mm selective inverse flow probe (FISEI, 120 µl active volume) with z-gradient. Specimens were automatically delivered to the spectrometer via flow injection. The acquisition temperature was set to 300°K. A standard one-dimensional 1H-NMR pulse sequence with suppression of the water peak (NOESYPREAST) was used: RD – P(90°) – 4 µsec – P(90°) – tm – P(90°) – acquisition of the free induction decay (FID). The non-selective 90° hard pulse P(90°) was adjusted to 9.4 µsec. The relaxation delay (RD), the mixing time (tm), and the acquisition time were set to 4 sec, 100 msec, and 3.96 sec, respectively, resulting in a total recycle time of ~8.0 sec. Low-power continuous-wave irradiation on the water resonance at an field strength of ~25 Hz was applied during RD and tm for pre-saturation. After application of 4 dummy scans, 32 FIDs were collected into 32768 (32K) complex data points using a spectral width of 20.689 parts per million (ppm). FIDs were multiplied with an exponential function corresponding to a line broadening of 0.3 Hz before Fourier-transformation. Spectra were manually phase and baseline corrected and automatically referenced to the internal standard (TSP – 0.0 ppm) within TopSpin 1.3 (Bruker BioSpin).

*Metabolomics Measurements: Quality Control and Normalization of Metabolite Levels*

The Fourier-transformed and baseline-corrected NMR spectra were manually annotated by spectral pattern matching using Chenomx NMR Suite 6.1 (Chenomx Inc., Edmonton, Alberta, Canada) to deduce absolute urinary concentrations of 56 metabolites; subsequently, the NMR data was reduced to these metabolites. Similar to MS measurements, urinary dilution was accounted for by PQN normalization. Normalized metabolite levels were once more log_2_-transformed.

**FIGURES**

**
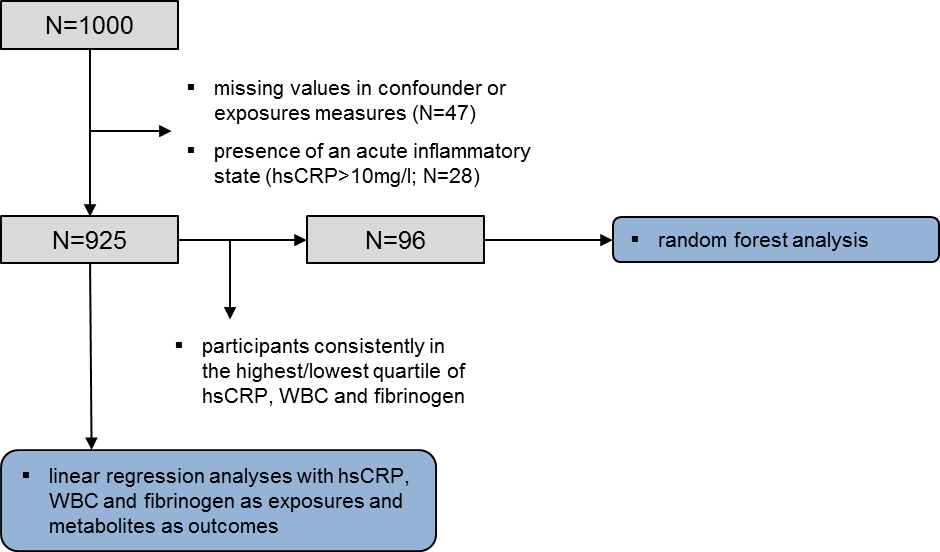
**

**Figure S1** Flowchart of the study compilation (grey) and subsequent statistical analysis (blue). hsCRP = high-sensitivity C-reactive protein; WBC = white blood cell count

***Classification of an advanced inflammatory state***


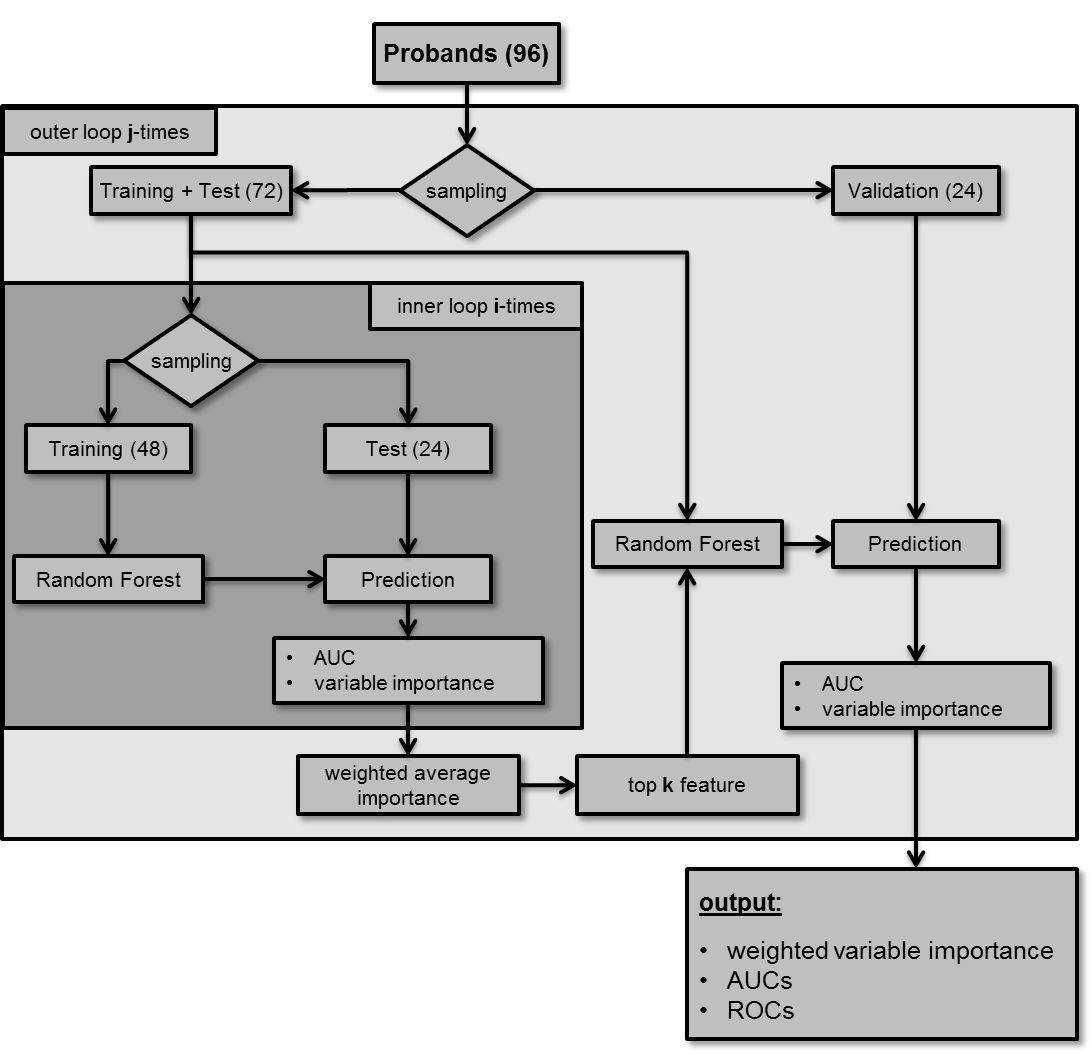


**Figure S2**. Flowchart of the classification procedure. Each outer loop started with splitting off the validation data from the remaining data that were further divided in a training set and a test set at each start of a training period. The training set was used to build a random forest (RF) exploiting all metabolites/clinical variables as features. Subsequently, the RF was used to predict the unseen test set and the performance was assessed by the area under the receiver operating characteristics curve (AUC) while feature importance was measured as the Gini index. Training was repeated on i different splits of data and an AUC-weighted mean Gini index was computed for all features. Afterwards, a new RF restricted to the top k features (those with the highest mean Gini index) was build. It was trained on the combination of training and test data and employed to classify the validation data. The described procedure was repeated j-times, once more yielding an AUC-weighted mean Gini index for feature importance. The final results of this procedure with i=20, j=20 and k=10 are shown in Figure 3 in the main text.


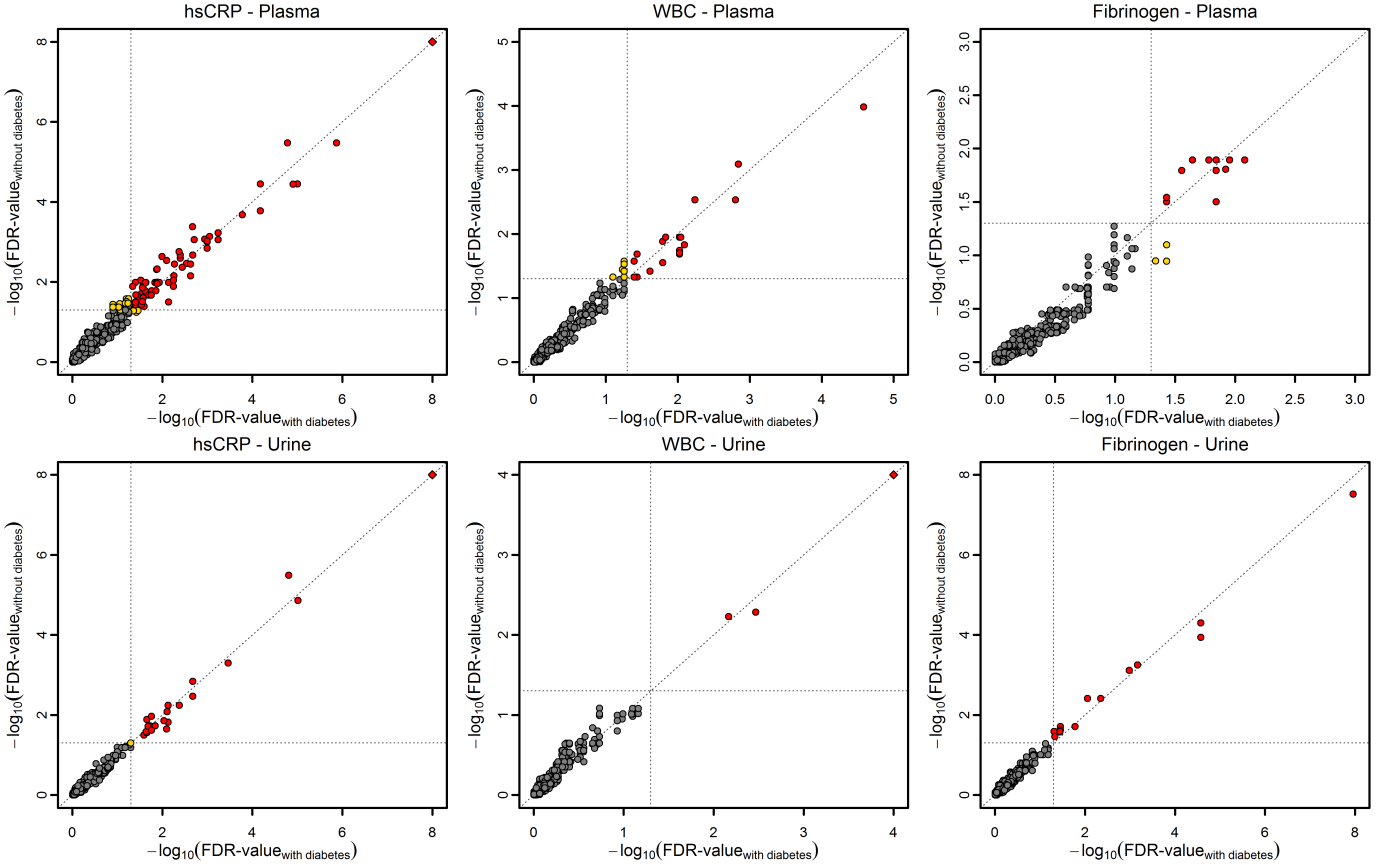


**Figure S3** Comparison of adjusted p-values (controlling the false discovery rate (FDR) at 5%) from linear regression analyses using either the whole population (with diabetes) or after exclusion of newly diagnosed diabetic subjects (without diabetes). Red dots indicate consistent significant results (FDR<0.05), whereas yellow dots indicate significant results in only one of the populations. Results for plasma metabolites are in the upper panel whereas results for urine metabolites are presented in the lower panel. hsCRP = high-sensitivity C-reactive protein


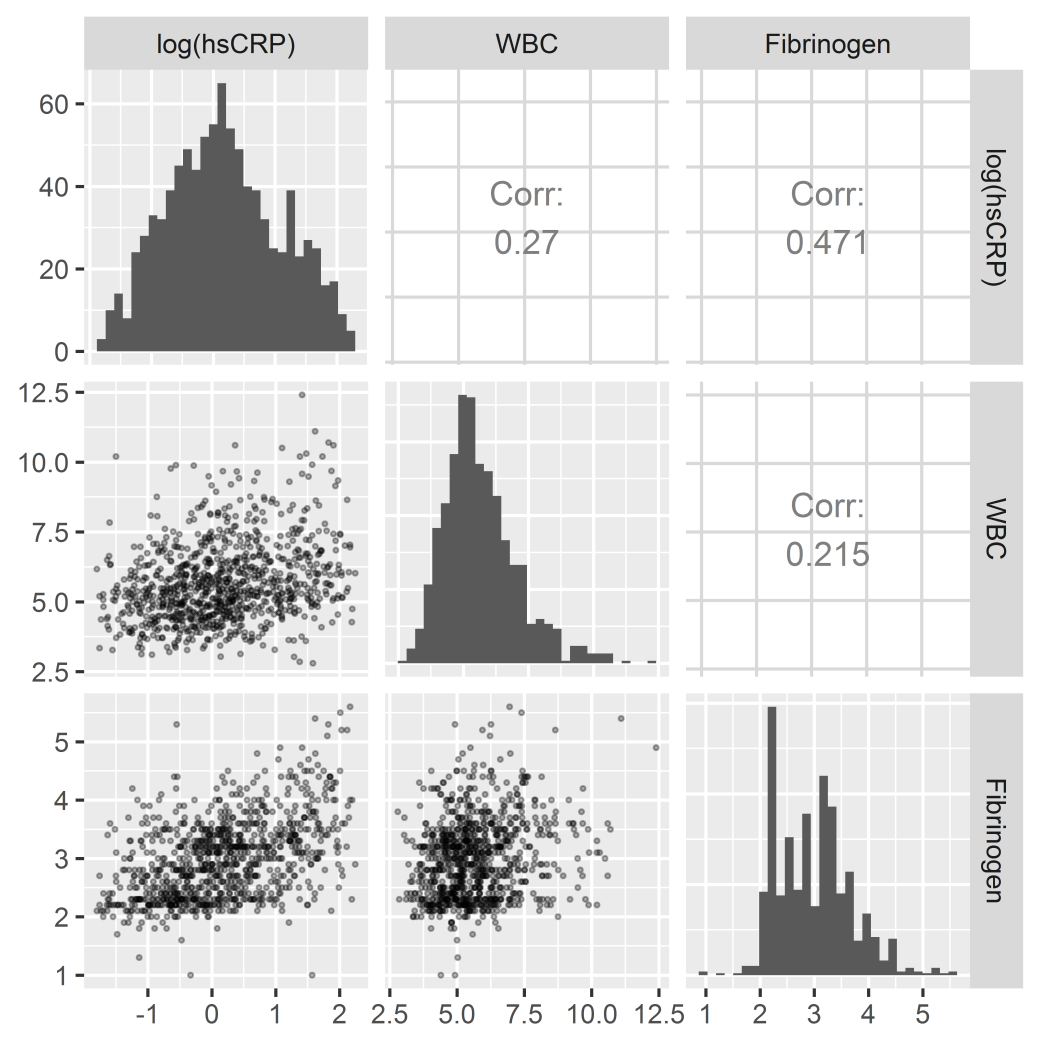


**Figure S4** Pairs plot of log-transformed high-sensitivity C-reactive protein [log(hsCRP)], white blood cell count (WBC) and fibrinogen levels. The lower triangle depicts individual scatter plots. The diagonal depicts histograms whereas in the upper triangle Pearson correlation coefficients (Corr.) are shown.

**REFERENCES**

1. Evans, A.M., et al., *Integrated, nontargeted ultrahigh performance liquid chromatography/electrospray ionization tandem mass spectrometry platform for the identification and relative quantification of the small-molecule complement of biological systems.* Anal Chem, 2009. **81**(16): p. 6656-67.

2. Lawton, K.A., et al., *Analysis of the adult human plasma metabolome.* Pharmacogenomics, 2008. **9**(4): p. 383-97.

3. Dieterle, F., et al., *Probabilistic quotient normalization as robust method to account for dilution of complex biological mixtures. Application in 1H NMR metabonomics.* Anal Chem, 2006. **78**(13): p. 4281-90.

4. Filzmoser, P., R. Maronna, and M. Werner, *Outlier identification in high dimensions.* Computational Statistics and Data Analysis, 2008. **52**(3): p. 1694-1711.
